# Recovery Potential of a Western Lowland Gorilla Population following a Major Ebola Outbreak: Results from a Ten Year Study

**DOI:** 10.1371/journal.pone.0037106

**Published:** 2012-05-23

**Authors:** Céline Genton, Romane Cristescu, Sylvain Gatti, Florence Levréro, Elodie Bigot, Damien Caillaud, Jean-Sébastien Pierre, Nelly Ménard

**Affiliations:** 1 UMR 6553, ECOBIO: Ecosystems, Biodiversity, Evolution, CNRS/University of Rennes 1, Biological Station of Paimpont, Paimpont, France; 2 School of Biological, Earth and Environmental Sciences, University of New South Wales, Kensington, Australia; 3 Université de Saint-Etienne, Equipe Neuro-Ethologie Sensorielle, ENES/CNPS CNRS UMR 8195, Saint-Etienne, France and UMR 8195, Centre National de la Recherche Scientifique, Centre de Neurosciences Paris-Sud, France; 4 Section of Integrative Biology, University of Texas at Austin, Austin, Texas, United States of America; 5 UMR 6553, ECOBIO: Ecosystems, Biodiversity, Evolution, CNRS/University of Rennes 1, Rennes, France; Australian Wildlife Conservancy, Australia

## Abstract

Investigating the recovery capacity of wildlife populations following demographic crashes is of great interest to ecologists and conservationists. Opportunities to study these aspects are rare due to the difficulty of monitoring populations both before and after a demographic crash. Ebola outbreaks in central Africa have killed up to 95% of the individuals in affected western lowland gorilla (*Gorilla gorilla gorilla*) populations. Assessing whether and how fast affected populations recover is essential for the conservation of this critically endangered taxon. The gorilla population visiting Lokoué forest clearing, Odzala-Kokoua National Park, Republic of the Congo, has been monitored before, two years after and six years after Ebola affected it in 2004. This allowed us to describe Ebola's short-term and long-term impacts on the structure of the population. The size of the population, which included around 380 gorillas before the Ebola outbreak, dropped to less than 40 individuals after the outbreak. It then remained stable for six years after the outbreak. However, the demographic structure of this small population has significantly changed. Although several solitary males have disappeared, the immigration of adult females, the formation of new breeding groups, and several birth events suggest that the population is showing potential to recover. During the outbreak, surviving adult and subadult females joined old solitary silverbacks. Those females were subsequently observed joining young silverbacks, forming new breeding groups where they later gave birth. Interestingly, some females were observed joining silverbacks that were unlikely to have sired their infant, but no infanticide was observed. The consequences of the Ebola outbreak on the population structure were different two years and six years after the outbreak. Therefore, our results could be used as demographic indicators to detect and date outbreaks that have happened in other, non-monitored gorilla populations.

## Introduction

Infectious diseases are increasingly recognized as a major threat to biodiversity, along with habitat loss and degradation, and climate change [1]–[4]. Factors influencing the emergence and propagation of infectious diseases are relatively well known in humans and domestic animals [1], [5], [6]. However, the occurrence of disease outbreaks and their consequences on wildlife remain insufficiently documented [7]. Several studies suggest that infectious diseases are emerging at an increasing rate in wildlife populations, as a result of closer contact between human populations and domestic and wild animals, changes in climate, and/or global land use. Such factors can contribute to the expansion of vectors' ranges [1], [8]–[13].

Emerging infectious diseases have been responsible for several cases of dramatic decline of wildlife populations (howler monkeys *Alouatta guariba clamitans* and *A. caraya* infected by Yellow fever [14], western lowland gorillas *Gorilla gorilla gorilla* infected by Ebola virus [15], [16]). In some cases the decline has led to population extinctions (black-footed ferrets *Mustela nigripes* infected by the canine distemper virus [17]; a range of amphibian species infected by *Batrachochytrium dendrobatidis* [18]) or even species extinction (snails *Partula turgida* affected by steinhausiosis [19], see also refs. [1] and [4] for reviews). Measuring the demographic consequences of disease outbreaks is often difficult because pre-epidemic data are rarely available, due to the unpredictable nature of disease outbreaks [20]–[23].

Several cases of population decline caused by infectious diseases have been documented in primates (see ref. [12] for a review in free-living primates). As human populations increase in size, human-primate contact becomes more frequent, increasing the probability of human-primate pathogen transmission [12]. The Ebola virus is a typical example of such a pathogen. Since the first known human case in 1976 [24], it has become a major threat to both biodiversity and human health. Ebola outbreaks have caused severe declines in chimpanzee and gorilla populations in Central Africa [15], [16], [25]–[27]. Detailed studies of the impact of Ebola on gorillas have been conducted on two populations from the Republic of the Congo (RC): the Lossi sanctuary population, where Ebola outbreaks were recorded in 2002 and 2003 [15], and the large gorilla population of Lokoué, Odzala-Kokoua National Park (377 individually identified gorillas [28]), affected in 2004 [16]. These two outbreaks resulted in mortality rates estimated at around 90–95%. During the Lokoué outbreak, individuals living in groups (mostly females and immature individuals) were more heavily affected than solitary males, highlighting the cost of sociality in terms of disease risk [16]. The dramatic impact of Ebola led the International Union for Conservation of Nature (IUCN) to upgrade the status of western gorillas from “endangered” to “critically endangered”.

Here we describe the demographic changes observed in the Lokoué gorilla population during the six years following the Ebola outbreak, based on analysis of demographic data collected beginning in 2001. As Ebola disproportionately affected group-living individuals, it had an immediate, marked effect on the structure of the population. In particular, the proportion of solitary males (silverbacks or blackbacks) increased [16]. We expected the social structure of the post-Ebola population to tend to go back to the normal, pre-Ebola structure. We hypothesized that in a relatively short period, the surplus of young silverbacks would tend to emigrate from the population to seek adult females, whereas unknown adult males and females would immigrate to form new groups. Secondly, we expected birth events to occur in the new groups, and thus the total population size to increase again. Below, we examine these hypotheses, and provide a detailed description of the post-Ebola population dynamics. We use our results to discuss the potential for recovery of the large gorilla population that was affected by Ebola.

## Materials and Methods

### Ethics statement

This research complied with the ethic guidelines of the CNRS/University of Rennes. This was an observational study. Observers remained quiet in a platform during observations and never tried to approach gorillas. Permission for this study was obtained from Odzala-Kokoua National Park (OKNP) and the Ministry of the Sustainable Development, the Forest Economy, and the Environment of the Republic of the Congo.

### Study site and duration

The study gorilla population was observed in the 4 hectare (ha) swampy clearing of Lokoué (00°54′23″N; 15°10′33″E). Forest clearings in Odzala-Kokoua National Park attract numerous large mammals, which feed on their herbaceous vegetation. The clearing vegetation has high mineral contents that gorillas do not find in food plants from the surrounding forest [29] (see also ref. [28] for a description of the Lokoué site). The camp was located 5 km away from the clearing, which minimized the disturbance to Lokoué gorillas. The gorilla population visiting the clearing was monitored between 2001 and 2010.

Observations were conducted during four periods, totaling 1215 observation days and 10,965 hours (Table 1): (1) before the Ebola outbreak, (2) during the Ebola outbreak (these data were only used to describe changes that concerned units known both after and before the outbreak), (3) two years after the Ebola outbreak, and (4) six years after the Ebola outbreak.

**Table 1:** Observation time and presence of gorillas on the Lokoué clearing during each period of the study.

		Observation		Gorilla presence
Period	Dates	N_0_ of days	N_0_ of hours	% of days
LBE	04-2001 to 09-2002	380	3668	94.5
LE	12-2003 to 11-2004	238	2042	66.4
LAE1	12-2004 to 05-2006	344	3110	41.3
LAE2	06-2008 to 04-2010	253	2145	22.9

LBE: Lokoué before Ebola, LE: Lokoué during Ebola, LAE1: Lokoué 2 years after Ebola, LAE2: Lokoué 6 years after Ebola.

### Observations

Gorillas were observed from a 4-meter-high platform located at the edge of the clearing. The distance between the gorillas and the platform varied between 5 and 200 m (the approximate diameter of the clearing). The presence of observers in the platform did not seem to alter the behavior of the gorillas. During observation periods, the clearing was monitored daily, from approximately 7:00 a.m. to 4:30 p.m., by one or two observers using a Kowa spotting scope (20×60), and either a video camera (Sony Handycam Digital 8 DCR-TRV 620E) or a Canon EOS 40D digital camera equipped with a 600 mm lens and a Teleplus 2× converter. Gorillas were individually identified using a combination of physical features: shape of the face (supraorbital torus, nostrils, wrinkles), shape of the sagittal crest for silverbacks, nose-print, scars, physical handicaps, general body shape and hair color patterns [28]. A catalogue of photos and sketches was created and used to compare identifications made by different observers.

Sex and age-class were determined following physical and behavioral criteria as described in ref. [16], [28], and age was derived from age classes as follows, using results from ref. [30]: infants (≤4 years old), juveniles (4 to 7.5 years old), subadults (7.5 to 10 years old for females, 7.5 to 11 years old for males), adult females (≥10 years old), adult males (young adult males or “blackbacks”: 11 to 14 years old; mature males or “silverbacks”: ≥14 years old). Here, the term “immatures” refers to females and males up to 10 and 11 years old, respectively. The assignment of a reliable birth date for infants born during our study was not always possible due to the large time lag (sometimes several months) between consecutive visits of gorilla units. We estimated the age of infants with unknown birth dates by comparing their morphology with that of individuals of known age. Gorillas' strong sexual dimorphism allowed us to easily determine the sex of adult individuals; but inconspicuous genitals of immature individuals rendered their sexing problematic. Immatures could only be occasionally sexed as they bowed to drink or to lick the soil.

Gorilla social units were categorized as solitary gorillas, breeding groups (BG, including adults of both sexes), and non-breeding groups, which did not include adult females and were typically composed of immature individuals and blackbacks [28], [31]. The individuals of each unit were identified during each visit.

The Lokoué population is not isolated. It is an open population, included in the much larger, continuous population ranging from the coast of Gabon to Central African Republic. For each of the three study periods, we therefore arbitrarily defined the Lokoué population as the set of social units observed on the clearing during the period. This definition assumes that the units visiting the clearing are representative of the population ranging in the vicinity of the clearing. Owing to the cumulative curves of gorilla unit numbers against number of days of observation of gorillas, we assumed that the three study periods were long enough to study changes in the composition and dynamics of the gorilla population visiting the Lokoué clearing (Figure S1). Statistical analyses comparing the structure of the population for the different study periods were performed using the group composition recorded the last time each group was observed. An individual was considered as an immigrant if it had never been observed before. Gorillas that disappeared between the two post-Ebola periods were considered as emigrants. As individuals who died fell into this category, the number of emigrants was likely slightly overestimated. However this bias can be assumed to be relatively small given gorillas' extended lifespan.

Birth rates were calculated for each period by dividing the number of newborns observed during the study period by the number of adult female-years, as in ref. [32] (*e.g.* one adult female with three months between its first and its last observation during the study period represents 0.25 adult female-years).

Emigration and immigration events were recorded between the Lokoué population pre- Ebola and two years post-Ebola, as well as between two and six years post-Ebola. All observed transfers of individuals between known groups were also recorded during the entire study.

### Statistical analyses

Generalized linear models (GLMs) with quasi family and variance proportional to the cube of the mean were used to compare count data between study periods. The quasi family was used to account for the overdispersion of the response variables. Modeled count data included: size of breeding groups and number of adult females per breeding groups. GLMs with binomial error and *logit* link function were used to model the following proportions: proportion of adult females and immature individuals *vs.* adult males, sex ratio of mature individuals (females/males), proportion of females with infants, birth rate and proportion of groups *vs.* solitary individuals. The effect of the study period on the proportion of individuals living in groups was tested using a binomial GLM, by coding the response variable as 1 for group-living individuals and 0 for solitary individuals. The effect of the study period on the different response variables was tested by analyzing the model deviances using *F*-tests (quasi likelihood models) and Chi-square tests (binomial models). All tests were performed using a type I error equal to 0.05. If an *F*-test was significant, post-hoc *t*-tests of the significance of the coefficient corresponding to each level of the independent categorical variable were performed after Bonferroni correction using function adjust.esticon() from the “RVAideMemoire” R package [33]. If a Chi-square test was significant, pairwise comparisons of the proportions were performed using the R function pairwise.prop.test(). If the post-hoc *t*-test was not significant while the global deviance test was, we considered that the difference between the two extreme values of the tested variable was significant. All statistical analyses were performed using R 2.11.1 [34].

## Results

### Changes in the Lokoué population structure after the Ebola outbreak

**Figure 1:**
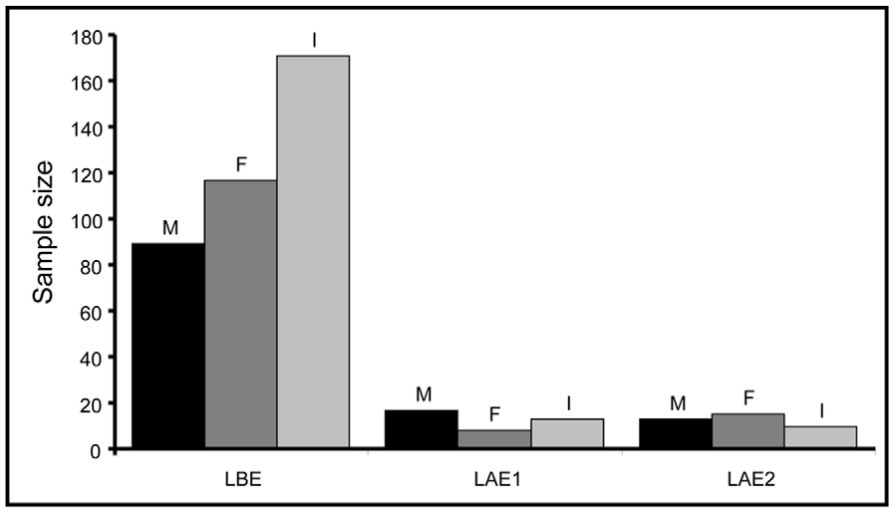
Number of adult males (M), adult females (F) and immatures (I) identified during the study periods. LBE: Lokoué before Ebola, LAE1: Lokoué 2 years after Ebola, LAE2: Lokoué 6 years after Ebola (see details in Table 1).

The gorilla population decreased from 377 known individuals before Ebola, to 38 known individuals two years after Ebola, and 40 known individuals six years after Ebola (Figure 1). The proportion of adult females and immatures varied significantly between the three study periods (binomial GLM, χ^2^ = 9.794, df = 2, *P* = 0.007). It dramatically decreased between the period pre-Ebola and two years after the Ebola outbreak (*t*-test, Bonferroni correction: df = 5; *P* = 0.024). The number of adult males and immatures slightly decreased between two years and six years post-Ebola (due to a decrease in the number of blackbacks and subadults) while the number of adult females doubled (not tested, see Figure 1).

**Figure 2:**
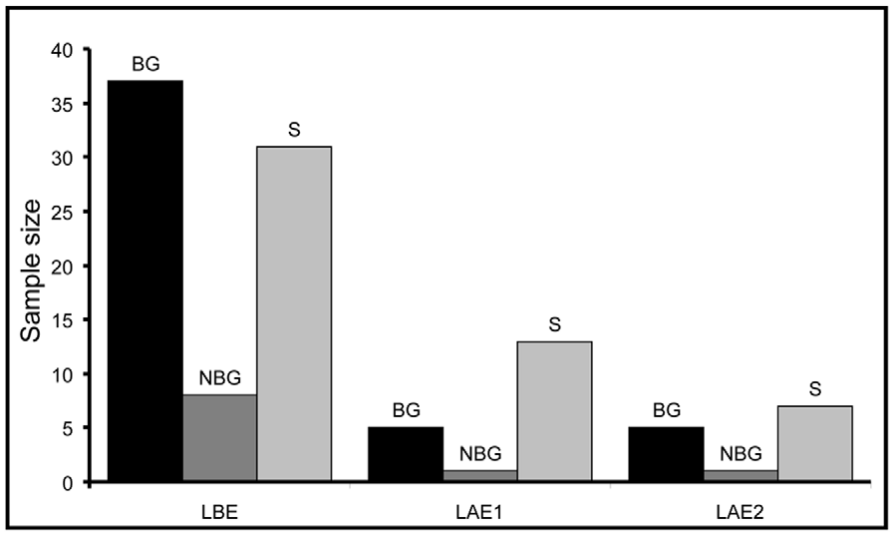
Number of breeding groups (BG), non-breeding groups (NBG) and solitary individuals (S) identified during the study periods. LBE: Lokoué before Ebola, LAE1: Lokoué 2 years after Ebola, LAE2: Lokoué 6 years after Ebola (see details in Table 1).

The number of social units dramatically declined between the period pre-Ebola and two years post-Ebola, particularly the number of groups, which fell from 45 to six. However, the proportion of groups among social units did not differ significantly between the three periods (binomial GLM, χ^2^ = 4.978, df = 2, *P* = 0.083; Figure 2). The number of units visiting the clearing decreased from 19 two years post-Ebola to 13 six years post-Ebola, which may explain the decrease in the percentage of observed days with gorillas (Table 1).

The proportion of individuals living in groups varied significantly between the three study periods (binomial GLM, χ^2^ = 19.089, df = 2, *P*<0.001). It decreased significantly, by a factor of 1.4, between the period pre-Ebola and two years post-Ebola (92% *vs.* 66%; *t*-test, Bonferroni correction: df = 5; *P*<0.001). Six years post-Ebola, it had increased compared to two years post-Ebola (82.5%) but the variation was not significant (t-test Bonferroni correction: df = 5; *P* = 0.460). The important decrease of the sample size after the outbreak may explain the non-significance of this latter result [35]. The proportion of individuals living in groups did not significantly differ between the period pre-Ebola and six years post-Ebola (*t*-test, Bonferroni correction: df = 5; *P* = 0.300). More than 84% of the solitary individuals were silverbacks or blackbacks, irrespective of the period. We recorded two solitary subadults two years post-Ebola and a unique case of a solitary adult female six years post-Ebola (one visit).

**Figure 3:**
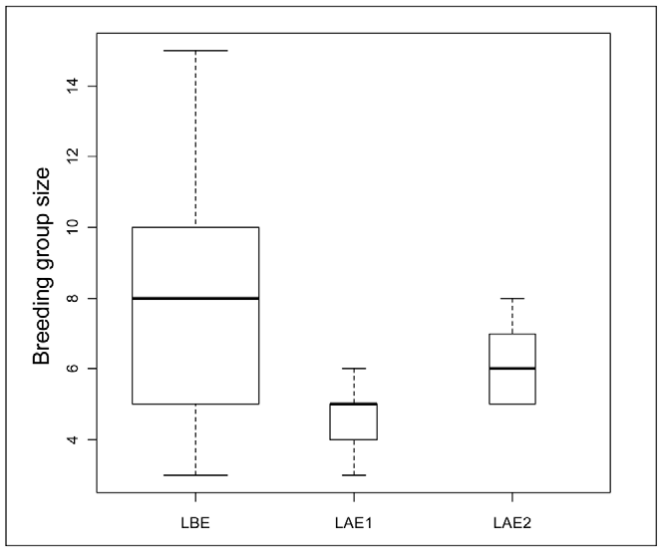
Boxplots of breeding group sizes during the study periods. LBE: Lokoué before Ebola, LAE1: Lokoué 2 years after Ebola, LAE2: Lokoué 6 years after Ebola (see details in Table 1). Bold horizontal lines are median values. The width of the boxes varies in proportion to the square roots of the number of observations in the groups. The ends of the vertical lines indicate the minimum and maximum values.

**Figure 4:**
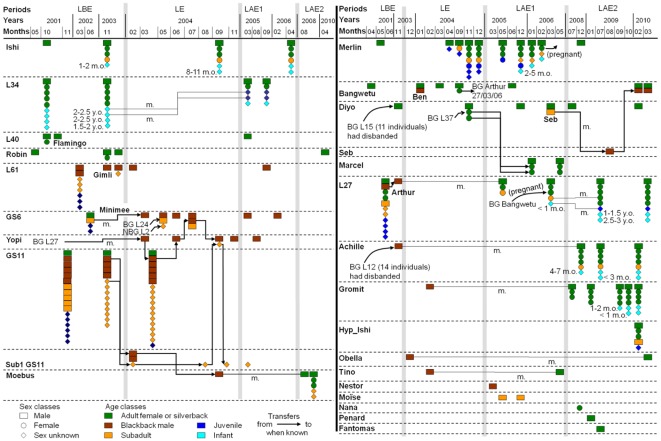
Dynamics of the units during the study period (2001–2010). Only the units observed after the Ebola outbreak are included. The unit compositions are given at the first visit on the clearing, for every change in composition, and at the last visit. Only the months and years when any change in a unit composition occurred are mentioned. The names of units or individuals are in bold. BG: Breeding group; NBG: non-breeding group. m.: maturation of an individual during the period indicated by a line. m.o. (months old) or y.o. (years old): estimates of the age of infants at the date of observation. The grey bars distinguish between the study periods. LBE: Lokoué pre-Ebola period: April 2001 to November 2003, LE: Lokoué during Ebola period: December 2003 to December 2004, LAE1: Lokoué post-Ebola period 1: December 2004 to May 2006 and LAE2: Lokoué post-Ebola period 2: June 2008 to April 2010.

The size of the breeding groups and the number of females they included varied significantly between the three study periods (group size: quasi likelihood GLM, *F*_2,44_ = 6.225, *P* = 0.004; number of females: quasi likelihood GLM, *F*_2,44_ = 3.671, *P* = 0.034). These quantities decreased significantly, by a factor of 1.8 and 2, respectively, between the period pre-Ebola and two years post-Ebola (group size: 8.2 *vs* 4.6; *t*-test, Bonferroni correction: df = 40, *P*<0.001; Figure 3; number of females: 3.2 *vs* 1.6; *t*-test, Bonferroni correction: df = 40, *P* = 0.001; Table 2), and did not significantly vary between two and six years post-Ebola (group size: *t*-test, Bonferroni correction: df = 8, *P* = 0.451; number of females: *t*-test, Bonferroni correction: df = 8, *P* = 0.300). Nevertheless, the mean breeding group size and the mean number of adult females per group observed six years post-Ebola no longer significantly differed from the period pre-Ebola (group size: *t*-test Bonferroni correction: df = 8, *P* = 0.207; number of females: *t*-test Bonferroni correction: df = 8, *P* = 1). Interestingly, in contrast to Lokoué before the outbreak, no BGs included blackbacks two or six years after the Ebola outbreak (Table 2 and Figure 4).

The mean size for non-breeding groups was 5.5 (2–15) individuals in the pre-Ebola Lokoué population. Non-breeding groups before Ebola were composed of all classes of immatures except infants, and most of the non-breeding groups were accompanied by a silverback. Only one non-breeding group comprising two individuals was observed two years post-Ebola (one silverback and one subadult male) and six years post-Ebola (one silverback and one blackback, Figure 4).

### Demography and dynamics of the Lokoué population after the Ebola outbreak

We identified 32 different units after the Ebola outbreak (Figure 4). Among the 377 individuals known in Lokoué before Ebola, only 22 were observed again after the outbreak. Among the 37 breeding groups known before the Ebola outbreak, only two were observed two years post-Ebola but were not seen again six years post-Ebola (Ishi, L34; Figure 4). Four other BGs (L40, Robin, L15, L12) disbanded before the outbreak, when their silverbacks and one blackback were observed as solitary males. Another BG (L27) was not seen after the outbreak, but two of its blackbacks were observed as solitary individuals (Yopi, Arthur). Among the eight non-breeding groups known in Lokoué pre-Ebola, none was observed after the outbreak. Only four individuals (three blackbacks and one subadult) from these non-breeding groups were observed again, as solitary individuals. Among the 31 solitary individuals known in Lokoué pre-Ebola, six were observed two years post-Ebola. Among these six, one was not seen again six years post-Ebola (Flamingo).

#### Breeding group instability

During the outbreak, three silverbacks (Merlin, Bangwetu, Diyo, see Figure 4) observed as solitary individuals before the outbreak formed breeding groups. In addition to their silverback, these three BGs were composed of: two adult females plus four immature individuals, one adult female, and two adult females, respectively. Each of these BGs disbanded during the two years after the Ebola outbreak, with a tenure length of less than two years. Two of their five females were not seen again. All three males were already mature in 2001 (>14 years old). Therefore, two of them were at least 17 years old when they held their BGs. We do not know whether they had previously formed BG's or not. The last one (Diyo) had previously lived in a BG of 11 individuals and had lost his tenure before the Ebola outbreak. The presence of one subadult among the progeny of this BG in 2001 suggested that the group had existed for at least 7–8 years. Consequently, we estimated that Diyo was more than 24 years old when he formed his second BG in 2004.

#### Formation of new breeding groups

After the outbreak, we observed the formation of five BGs and the immigration of one BG (Hyp-Ishi) in the six-year period post-Ebola (Figure 4). Three of the newly formed BGs included previously-known solitary males (Arthur two years post-Ebola, Achille and Moebus six years post-Ebola) and two included immigrant solitary males (Marcel two years post-Ebola, Gromit six years post-Ebola). Among the six new BGs, four were formed from young silverbacks who had reached their maturity during the course of our study. They formed their first BG at an estimated age of 15 (Arthur), 16 (Moebus), and 18 years (Achille, Gromit). Only one of the 20 females in these BGs was known before the outbreak.

#### Female transfer

One female with her infant (4 to 7 months old) joined the silverback Achille who was solitary three months before and was unlikely to have sired this offspring. In addition, an immigrating female left Bangwetu while she was pregnant, and subsequently joined Arthur's new breeding group, where she gave birth two years post-Ebola. The infant of that female survived to become a juvenile in Arthur's group during the observation period six years after the Ebola outbreak, despite the fact that Arthur was likely not his father.

#### Infant recruitment

During both post-Ebola periods, average birth rates were 0.31 births/female-year and 0.38 births/female-year, respectively, compared to 0.32 births/female-year in the pre-Ebola population. No significant difference was found between these birth rates (binomial GLM, χ^2^ = 0.172; df = 2; *P* = 0.918). A total of 11 infants were observed in breeding groups after the outbreak. Two were born two years post-Ebola (in Arthur's and Merlin's groups), four were born six years post-Ebola (in Achille's and Gromit's groups) and two were born between two and six years post-Ebola (in Arthur's group, Figure 4). The three remaining infants were born just before or during the outbreak (Ishi's and L34 groups, Figure 4). The proportions of adult females with infants did not significantly differ between periods (binomial GLM, χ^2^ = 2.943; df = 2; *P* = 0.230). At the end of the study, nine females out of 14 were potential future breeders (eight adult females without infants and one subadult female). Three of the six new BGs produced offspring (Achille's, Arthur's, and Gromit's groups), and the juvenile observed in the Hyp-Ishi group was potentially born after the Ebola outbreak (Figure 4).

#### The fate of the unmated males

Among the 13 solitary males observed two years post-Ebola, eight were known before Ebola and five were immigrants (Figure 4). Eight of them were not seen again six years post-Ebola. These comprised four blackbacks and one young silverback of 13–14 years, one silverback of at least 18 years, and two subadults. Among the seven solitary individuals in the population six years post-Ebola, four were known individuals and three were immigrants (two silverbacks and one adult female). During the two years post-Ebola period, Diyo formed a non-breeding group with an immigrant subadult male (Seb). At the beginning of the six-year period post-Ebola, Diyo and Seb became again solitary, and Seb subsequently joined Bangwetu, forming another non-breeding group. Two solitary blackbacks became silverbacks during the post-Ebola period and remained solitary until the end of our study, when they were 14 and 18 years old.

Five males disappeared between two and six years post-Ebola. They would have been young silverbacks six years after the outbreak (14–16 years old). At the end of the study, among the seven mature males living in non-BG, at least four were more than 23 years old (one of them, Diyo, being at least 30 years old in 2010). One silverback had an estimated age of 18 years and the two remaining silverbacks were of unknown age.

#### To summarize

Although the size of the Lokoué population remained stable during the six years after the Ebola outbreak, the population structure was highly dynamic. After the outbreak, only two pre-Ebola BGs remained. However, the population post-Ebola shows signs of recovery, with the formation of six new breeding groups including mostly immigrant females (95%, N = 20) and young silverbacks. In contrast, the percentage of immigrant males was lower (silverbacks: 41%, N = 17; blackbacks: 43%, N = 7). Only three adult females and 11 silverbacks or blackbacks known before Ebola were observed again two years post-Ebola. Seven of these males were observed again six years after the outbreak, but none of the adult females were seen during this period. Most post-Ebola groups were small and included a small number of juveniles and subadults. Blackbacks, in particular, were completely absent from the BGs post-Ebola. We noticed that the estimated age at which a male formed his first BG varied from 15 to 18 years. The reconstruction of BGs was associated with birth rates similar to those observed in the population before the Ebola outbreak. Between two and six years post-Ebola the composition of the population appeared particularly unstable. Among 25 adults (17 males and eight females) observed two years post-Ebola, 11 (nine males and two females) were observed again six years post-Ebola. Among 28 adults (13 males and 15 females) observed six years post-Ebola, 16 (four males and 12 females) had immigrated after the two-year period post-Ebola. Therefore, we observed a change of 76% in the adult population from two to six years after the Ebola outbreak. If we consider the whole known population (infants born after outbreak excluded) of 52 individuals, only 23% were identical between the two periods. This led to a renewal of the population of about 59% (N = 32) between two and six years post-Ebola.

## Discussion

### Effects of an Ebola outbreak on a gorilla population

The present study shows that after an Ebola outbreak, the western lowland gorilla population of Lokoué slowly recovered the demographic structure of an unaffected population, as a result of migratory events, the formation of new BGs and the occurrence of new births.

**Table 2:** Composition of breeding and non-breeding groups in the Lokoué population before and after the Ebola outbreak.

		Group composition [mean number ± SD, (range)]						
Period	Group type	SB	AF	BB	Sub	Juv	Inf	NI
LBE	Breeding groups	1±0	3.2±1.8	0.2±0.7	1±1	0.8±1.1	2±1.3	0
	(n = 37)	(1-1)	(1–7)	(0–4)	(0–4)	(0–4)	(0–5)	
	Non-breeding groups	0.9±0.4	0	0.9±1.2	2.3±1.9	1.5±2	0	0
	(n = 8)	(0–1)		(0–3)	(0–5)	(0–6)		
LAE1	Breeding groups	1±0	1.6±0.5	0	0.6±0.5	0.4±0.9	1±0.7	0
	(n = 5)	(1-1)	(1–2)		(0–1)	(0–2)	(0–2)	
	Non-breeding groups	1	0	0	0	0	0	0
	(n = 1)							
LAE2	Breeding groups	1±0	2.8±0.8	0	0.4±0.5	0.4±0.5	1.2±1.1	0.4±0.9
	(n = 5)	(1-1)	(2–4)		(0–1)	(0–1)	(0–2)	(0–2)
	Non-breeding groups	1	0	1	0	0	0	0
	(n = 1)							

SB: Silverback, AF: Adult Female, BB: Blackback, Sub: Subadult, Juv: Juvenile, Inf: Infant, NI: Non Identified, LBE: Lokoué before Ebola, LAE1: Lokoué 2 years after Ebola, LAE2: Lokoué 6 years after Ebola (see details in Table 1).

Some of the changes in the structure of the Lokoué population after the outbreak were only observed temporarily, and had disappeared after six years. These short-term effects consisted of a decrease in the proportion of individuals living in groups, a decrease in the mean BG size, and a decrease in the mean number of adult females per BG. Six years post-Ebola these parameters did not differ anymore from those of other known western lowland gorilla populations, although the values remained slightly lower (proportion of individuals living in group: 82.5% at Lokoué *vs.* 93.9% at Mbeli [36] and 95.1% at Maya [37]; mean number of adult females in BGs: 2.8 at Lokoué *vs.* 3.5 at Mbeli [38] and 4 at Maya [37]).

Other effects of the outbreak on the structure of the population persisted after six years. These long-term effects included the absence of large breeding groups with all classes of immature individuals. Before Ebola, groups of different ages existed, including nascent, mature, and senescent groups (definition *sensu* [36]). By contrast, after the outbreak, BGs were predominantly newly formed and no BGs displayed the classical structure of mature groups (*i.e.*, including all age classes, especially blackbacks). Breeding groups contained fewer adult females and offspring than before the outbreak. It may be another decade before large mature and senescent groups are encountered at Lokoué.

A second long-term impact is the reduced population size. After the Ebola outbreak, only 40 different individuals were observed in 597 observation days, compared to a total number of 377 individuals observed in 380 days before the outbreak. The size of the Lokoué population remained stable six years after the outbreak. Two mechanisms could allow the Lokoué population to recover more rapidly: increased population growth (via increased birth rate and/or decreased mortality rate) and increased immigration. Compensatory changes in life-history traits can enable animal populations to recover from perturbations and have rescued populations from the brink of extinction [39]. Demographic responses to a reduction in population size include increased reproductive rates, earlier onset of sexual maturity, increased survival of some population classes, or increased recruitment rates [40]. However, while compensatory responses to changes in population abundance and density are known for species with a long lifespan [41], [42], such responses to disease outbreaks remain largely unexplored [43], [44]. So far we did not encounter compensatory responses in life-history traits in the Lokoué gorilla population after the Ebola outbreak. Instead, the instability of the Lokoué population in the post-Ebola period may have slowed down the growth of the population by inducing a reduction in the fecundity of females due to the repeated transfers of these females between groups [32] (see also below). As opposed to other study on western lowland gorillas, we did not find evidence that group instability increases the infanticide risk [32]. More time is needed to investigate whether compensatory responses in life history traits exist or not.

For immigration to play a significant role, the Lokoué population would need to be able to attract dispersing individuals. Indeed, animals rely partly on social information for choosing breeding sites [45], [46]. However, the Lokoué population has a low mating partner potential: all females are already included in nascent BGs with reproduction, the breeding potential of males is restrained to three silverbacks (four others are likely in their post reproductive period), and mature progeny is still lacking. Thus, the population might be limited to a first stage of slow internal growth, based on the maturation of BGs.

Another way for Lokoué to attract new units would be to present a superior ecological value compared to its surroundings. The forest area covered by the initial population is estimated at around 117 km^2^, according to gorilla density before Ebola (3.22 gorillas/km^2^ [47]). A study based on nest count surveys within a 4 km radius around the Lokoué clearing showed a reduction of 82% of gorilla density since 2004 [47], which was consistent with our findings from the Lokoué clearing. This area is now well below its carrying capacity, which might limit the risk of intra-specific competition, and it includes an attractive clearing. However, a possible recolonization of this site would depend on the existence of a source population in the surrounding areas and on the mobility of its social units. Some observations lead us to suspect that Ebola affected the surrounding populations (see below). Thus immigration could be limited by the lack of a source population. Finally, the home range size of a unit of western lowland gorilla is relatively stable, due to its predominantly frugivorous diet [48]–[51]. Social units are thus not expected to shift their home ranges to Lokoué.

### Reasons for the observed demographic effects after Ebola outbreak: behavioral strategies

The surviving males had to choose between staying in a population where females were rare and herded in a small, easily guarded BG, or emigrating in search of breeding opportunities. It seems that individuals with no immediate reproductive potential (blackbacks, subadults) chose to leave the Lokoué population, while young silverbacks remained to form new BGs with immigrant females. Old silverbacks remained solitary in the population. While Breuer and collaborators [30] proposed that adult silverback begins at 18 years of age (based on unpublished data that a male can acquire a female at the estimated age of 18 years at Mbeli), we observed at Lokoué that males can form their first BG from an age of 15 years. We do not know whether the discrepancy between the two studies resulted from the post-Ebola context at Lokoué or not. Nevertheless, these parameters of life-history should be taken into account in models of population dynamics and recovery capacity.

Adult females and immatures were subjected to a sudden destruction of their BG. This triggered them to rapidly seek the protection of a new silverback [52]. In the short term for female gorillas, protection seems to prevail over reproduction. Indeed, during Ebola, adult females formed BGs with three solitary silverbacks but only one gave birth before the groups disbanded. At least one of these silverbacks led BG previous to Ebola but had subsequently lost his status. He had a low head-crest size and poor musculature. Females seeking a new group are thought to avoid males with such morphological characteristics [53]. Aging males, likely excluded from reproduction, may show protection abilities towards adult females as they do towards immatures in non-breeding groups [31]. The low reproduction within these BGs and the early departure of the females suggest that the females did not choose the three silverbacks by sexual selection. This stage observed during the post-Ebola period, favoring protection over reproduction, was also reported in unaffected populations [32]. It is also similar to the dynamics of eastern gorillas observed after a demographic crash due to civil war [54].

By joining silverbacks, females benefit from their immediate protection against predators and can subsequently take advantage of inter-unit encounters to choose a silverback better suited for breeding. Among the five females mentioned above, three changed silverbacks during the post-Ebola period. One of these migrations led rapidly to a birth and this new BG persisted. A latency period for the selection of the breeding silverbacks could explain the fact that most births occurred more than nine months after Ebola, as already observed in unaffected populations when a group disbands [32]. Currently, four out of the five breeding silverbacks are 18 years old or younger. The presence in the population of young silverbacks that are high-quality candidates for sexual selection might also play a role in the stability of BGs, as it ensures an available breeding potential for adult females and thus allows them to remain in the population in the long term.

It is worth noting the observation of one female joining a BG with her infant (4 to 7 months old), and one pregnant female joining a BG where she gave birth. Both infants survived. In contrast with infanticidal behavior of mountain gorillas, our observations are consistent with those reported at Mbeli, where in four cases, dependant infants survived after mother/infant pairs transferred to new groups [32], [54]. Although these authors also suspected two cases of infanticide occurring in this population, their results and the present study underline that in western lowland gorillas, infanticide is not systematic [32]. More generally, there are behavioral and demographic features, which were already reported in unaffected western lowland gorilla populations, that may favor the recovery of the Lokoué population. These are: the presence of a single silverback within breeding groups, the protective role of post-reproductive silverbacks for females and vulnerable infants, and a certain tolerance of silverbacks towards infants they have not sired [28], [31], [32].

The context of the clearing, which potentially promotes immigration, could contribute to rapid changes in population structure. It remains to investigate if and to what extent this immigration could threaten the stability and the maturation of newly-formed breeding groups at Lokoué, slowing the recovery process. Further studies comparing the dynamics of the Lokoué population with that of other populations living in forests devoid of clearings are required to evaluate how important the clearing is to the recovery of the Lokoué population after the Ebola outbreak.

### Ebola and gorilla conservation

Infectious diseases can cause population extinction, particularly if they are combined with other factors such as small population size [55], [56]. As mentioned above, we estimate that the Lokoué population covers an area of approximately 117 km^2^. The impact of Ebola in the area beyond Lokoué is still not well understood. There are however some indications that Ebola might have affected the surrounding populations. For instance, the groups that immigrated to Lokoué after the outbreak were all nascent. Also, the Maya Nord population, located 52 km North West from Lokoué, had more than 400 individuals in 1996 [37] but only a few gorilla signs were detected in May and July 2004 (M. Douadi and DC, unpublished data). Recent nest count surveys were consistent with these observations, supporting a decrease in gorilla density in the northern part of the Park since 2005 [57]. The fact that new individuals are immigrating indicates that Lokoué is not surrounded by areas entirely devoid of gorillas, therefore it is not a small isolated population. Without additional environmental catastrophes (the risk of a new Ebola outbreak is now limited due to the low great ape density), it seems that we can expect the Lokoué population to recover. However, the extremely low gorilla density around Lokoué and the slow life history of this species will make this recovery process very slow. Demographic modeling studies will provide further information on the recovery potential of the Lokoué population.

In term of conservation, this study underlines that the preservation of large areas is critical. We cannot rely on protecting one population as it may be heavily impacted by stochastic dramatic event (*e.g.*, disease outbreaks in our case, fires or cyclones in other cases). We need instead to protect areas large enough to allow metapopulation dynamics to occur.

### Indicator of outbreaks

On the basis of this study, some indicators can be developed to detect populations that have been affected by the Ebola virus but for which no previous data are available. The modifications to the Lokoué population structure in the post-Ebola period (decrease of the proportion of individuals living in groups, of the BG size and the number of adult females they include) are indications of a recent outbreak (approximately a few years). Owing to the minimum time of 11 years (age of blackback) for a BG to reach the mature BG status, the absence of large mature and senescent groups is an indication of a dramatic event further in the past. A small gorilla population size (one of the long-term consequences of the Ebola outbreak in our study) cannot be considered as an indicator of a past outbreak, as it could be explained by many other factors including ecological conditions.

### Conclusion

On the basis of this study, we expect the Lokoué gorilla population to experience a first stage of slow growth, permitted by the maturation of current BGs. Sometime in the next few decades, a stage of exponential growth could be supported by the immigration of individuals attracted by the breeding potential of the population, which would in turn enhance the formation of new BGs. The same kind of two-step recovery scheme has been observed in a population of Venezuelan red howler *Alouatta seniculus* following habitat regeneration [58]. On the basis of the state of the Lokoué population six years after Ebola and the conditions of its recovery, we cannot expect the recovery to be less than the 60 years suggested by Walsh [27] for populations dramatically affected by Ebola. This time of recovery seems even optimistic due to the small size of the population six years after Ebola, the length of the sexual maturation (more than ten years for adult females) and the age at first BG (15–18 years for males) in western gorilla. Nonetheless, if sociality has a cost in case of Ebola outbreaks in gorilla populations [16], it also favors the recovery of the populations. Affected groups can either entirely disappear or leave some surviving but isolated adult females or immatures. The structure of the population spares solitary males, which are essential to protect surviving individuals and form new groups.

The importance of understanding the demographic consequences of Ebola, and thus the ability to recover, of an affected population of this long generation time and low birth-rate species, cannot be underestimated. Gabon and the Republic of the Congo harbor the world's largest studied gorilla populations. Along with urbanized zones, Ebola outbreaks are the major driver of the current distribution of great apes [27]. Given these two facts, understanding the effects of Ebola virus outbreaks that are widespread in both countries may be the only way to ensure this endangered species is protected for future generations.

## Supporting Information

Figure S1 **Cumulative number of gorilla units against the number of observation days with gorillas in the three study periods.** Green line: Lokoué before Ebola; blue line: Lokoué 2 years after Ebola; red line: Lokoué 6 years after Ebola; solid line: groups; dashed line: solitary individuals. None of the curves reaches an asymptote due to the continuous immigration of new units.
(TIF)
