# Supplementary figures and images for: Recovery Potential of a Western Lowland Gorilla Population following a Major Ebola Outbreak: Results from a Ten Year Study
Source: PLoS One. 2012 May 23;7(5):e37106. doi: 10.1371/journal.pone.0037106 (PMC3359368; doi:10.1371/journal.pone.0037106)

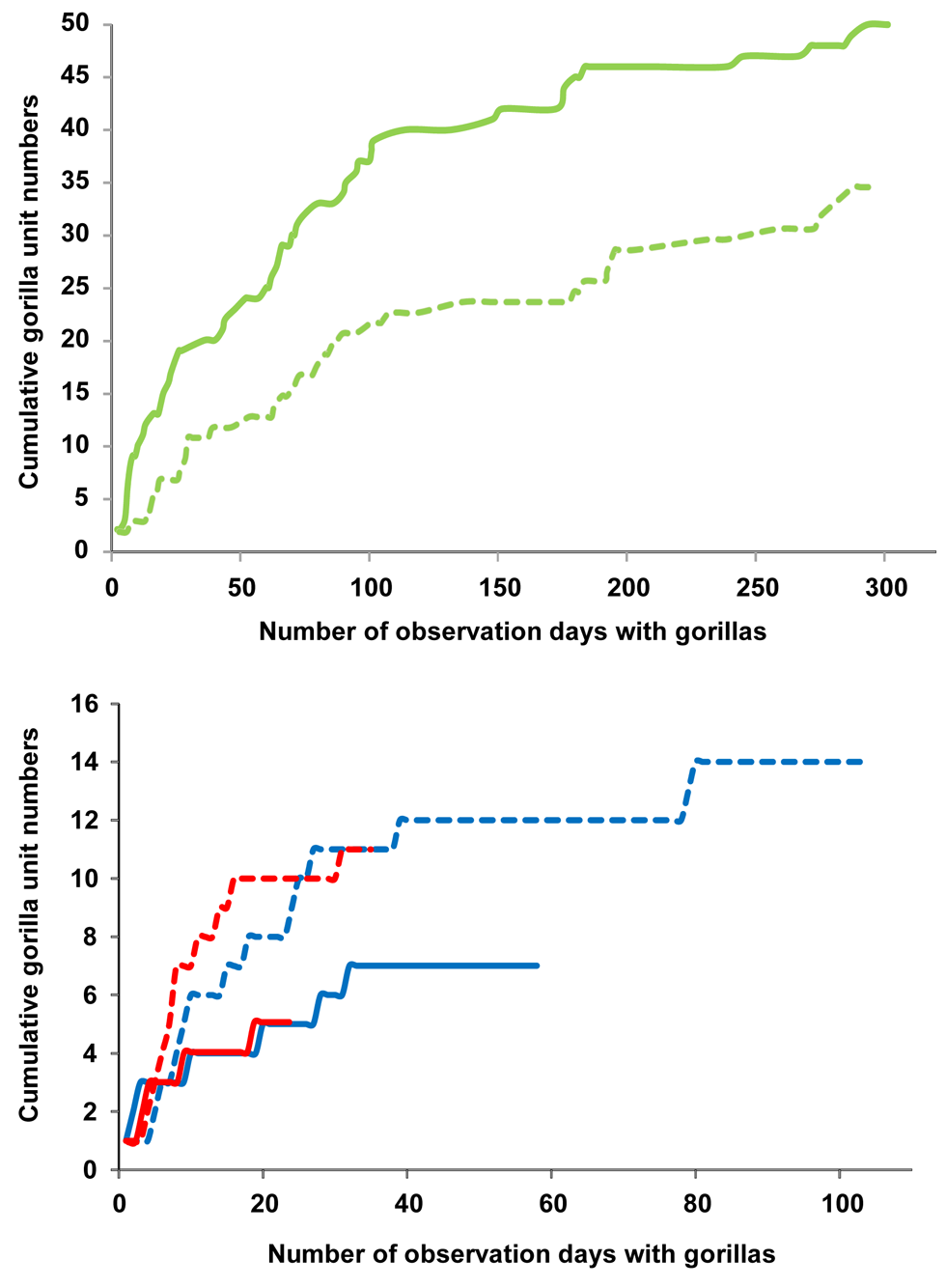

Supplement: Figure S1 — Cumulative number of gorilla units against the number of observation days with gorillas in the three study periods. Green line: Lokoué before Ebola; blue line: Lokoué 2 years after Ebola; red line: Lokoué 6 years after Ebola; solid line: groups; dashed line: solitary individuals. None of the curves reaches an asymptote due to the continuous immigration of new units. (TIF) [file pone.0037106.s001.tif]
